# Retraction: Cyclic AMP-response element regulated cell cycle arrests in cancer cells

**DOI:** 10.1371/journal.pone.0328370

**Published:** 2025-07-17

**Authors:** The PLOS One Editors

Following the publication of this article, concerns were raised with results presented in Figs 2–7. Specifically,

- The following panels appear similar:
  - Fig 2B cyclin B1 of this article [1] and Fig 1B Gapdh of [2] when rotated 180°
  - Fig 3B cyclin A and Fig 4B cyclin D1
  - Fig 3B CDK2 and Fig 4B CDK2 despite being used to represent different cell lines
- Bands in the following lanes appear more similar than would be expected from independent results:
  - Fig 2B actin lanes 1-2 and Fig 2B actin lanes 3-4
  - Each Fig 3B actin lane,
  - Fig 7C cyclin A lane 1 and Fig 7C lane 1,
  - Fig 7C cyclin A lane 2 and Fig 7C lane 4 when flipped horizontally,
  - Fig 7C cyclin A lane 4 and Fig 7C lane 3 when flipped horizontally,
  - Fig 7C cyclin D1 lanes 1-2 and Fig 7C actin lanes 3-4
- When adjusting the colour levels to visualize the background, there appear to be irregularities in the background of the following panels:
  - Fig 2B CDK2
  - Fig 3B cyclin B1
  - Fig 3B CDK4
  - Fig 4B actin
  - Fig 5C CDK4
  - Fig 6C cyclin D1
  - Fig 7C cyclin B1
  - Fig 7C CDK4

In response to these concerns, author JC stated that they did not consent to being included as an author and requested retraction of the article,

In light of the above unresolved concerns that question the reliability and integrity of these results, the *PLOS One* editors retract this article.

JC agreed with the retraction. PW, SH, FW, YR, MH, and XW either did not respond directly or could not be reached.
